# Exercise during and after neoadjuvant rectal cancer treatment (the EXERT trial): study protocol for a randomized controlled trial

**DOI:** 10.1186/s13063-017-2398-1

**Published:** 2018-01-12

**Authors:** Andria R. Morielli, Nawaid Usmani, Normand G. Boulé, Diane Severin, Keith Tankel, Tirath Nijjar, Kurian Joseph, Alysa Fairchild, Kerry S. Courneya

**Affiliations:** 1 Faculty of Kinesiology, Sport, and Recreation, University of Alberta, 1-113 University Hall, Van Vliet Complex, Edmonton, AB T6G 2H9 Canada; 2 Department of Oncology, University of Alberta and Cross Cancer Institute, 11560 University Avenue, Edmonton, AB T6G 1Z2 Canada

**Keywords:** Exercise, Cancer, Symptom management, Quality of life, Physical fitness, Physical activity

## Abstract

**Background:**

Standard treatment for locally advanced rectal cancer includes 5–6 weeks of neoadjuvant chemoradiotherapy (NACRT) followed by total mesorectal excision 6–8 weeks later. NACRT improves local disease control and surgical outcomes but also causes side effects including fatigue, diarrhea, hand-foot syndrome, and physical deconditioning that may impede quality of life (QoL), treatment completion, treatment response, and long-term prognosis. Interventions to improve treatment outcomes and manage side effects that are safe, tolerable and low-cost are highly desirable. Exercise has been shown to improve some of these outcomes in other cancer patient groups but no study to date has examined the potential benefits (and harms) of exercise training during and after NACRT for rectal cancer.

**Methods/design:**

The Exercise During and After Neoadjuvant Rectal Cancer Treatment (EXERT) trial is a single-center, prospective, two-armed, phase II randomized controlled trial designed to test the preliminary efficacy of exercise training in this clinical setting and to further evaluate its feasibility and safety. Participants will be 60 rectal cancer patients scheduled to receive long-course NACRT followed by total mesorectal excision. Participants will be randomly assigned to exercise training or usual care. Participants in the exercise training group will be asked to complete three supervised, high-intensity interval training sessions/week during NACRT and ≥ 150 min/week of unsupervised, moderate-to-vigorous-intensity, continuous exercise training after NACRT prior to surgery. Participants in the usual care group will be asked not to increase their exercise from baseline. Assessments will be completed pre NACRT, post NACRT, and pre surgery. The primary endpoint will be cardiorespiratory fitness (VO_2_ peak) at the post-NACRT time point assessed by a graded exercise test. Secondary endpoints will include functional fitness assessed by the Senior’s Fitness Test, QoL assessed by the European Organisation of Research and Treatment of Cancer, and symptom management assessed by the M.D. Anderson Symptom Inventory. Exploratory clinical endpoints will include treatment toxicities, treatment completion, treatment response, and surgical complications.

**Discussion:**

If the preliminary findings of EXERT are positive, additional research will be warranted to confirm whether exercise is an innovative treatment to maintain QoL, manage side effects, and/or improve treatment outcomes in rectal cancer patients.

**Trial registration:**

ClinicalTrials.gov, ID: NCT03082495. Registered on 9 February, 2017.

**Electronic supplementary material:**

The online version of this article (doi:10.1186/s13063-017-2398-1) contains supplementary material, which is available to authorized users.

## Background

**Fig. 1:**
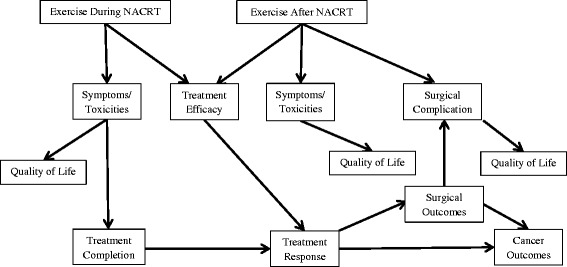
Proposed effects of exercise during and after neoadjuvant chemoradiotherapy in rectal cancer patients

Current standard treatment for locally advanced rectal cancer (stages II and III) includes long-course (5–6 weeks) neoadjuvant chemoradiotherapy (NACRT) followed by surgical resection using total mesorectal excision 6–8 weeks later [1]. When compared to postoperative chemoradiotherapy, NACRT improves local recurrences rates and may improve surgical outcomes in some patients [2, 3]. Unfortunately, NACRT causes acute toxicities including fatigue, diarrhea, hand-foot syndrome, hematologic toxicity, cardiotoxicity, and physical deconditioning that can cause declines in quality of life (QoL) [4] and may even impede treatment completion, treatment response, and long-term prognosis. Safe, tolerable, and low-cost interventions to manage these side effects and improve treatment outcomes in this clinical setting are highly desirable. We propose that an exercise training intervention initiated during NACRT in patients with rectal cancer may improve cardiorespiratory fitness, symptom management, QoL, treatment completion, treatment response, surgical complications, and possibly even survival (Fig. 1).

Exercise has been shown to manage some side effects and improve QoL in several cancer patient groups receiving adjuvant therapy [5]. Moreover, limited research has suggested that exercise during chemotherapy for some cancer patient groups may improve chemotherapy completion rates [6–8], treatment response [9], and even long-term survival [10]. Additionally, there is some evidence that pre-operative exercise may improve fitness and surgical outcomes in cancer patients [11]. Finally, preliminary evidence has suggested that exercise is feasible and safe in the neoadjuvant setting [12].

Despite the emerging evidence for the benefits of exercise in some cancer patient groups receiving some treatment protocols, only preliminary research has examined exercise in rectal cancer patients during and after NACRT. Similar to drug trials, exercise trials have demonstrated that research in one cancer patient/treatment group rarely generalizes to another cancer patient/treatment group [13]. Preliminary research suggests that exercise initiated immediately *after* NACRT is feasible and may improve cardiorespiratory fitness [14], which has prompted a phase II trial in this clinical space [15]. Furthermore, two phase I studies [16, 17], including one from our group [16], have demonstrated the preliminary feasibility and safety of exercise *during* NACRT for rectal cancer patients. Finally, one ongoing randomized controlled trial is examining the feasibility of an unsupervised walking program both during and after NACRT (ISRCTN62859294). Here, we propose the Exercise During and After Neoadjuvant Rectal Cancer Treatment (EXERT) trial which, to our knowledge, is the first phase II trial designed to examine the preliminary efficacy of exercise training in rectal cancer patients during and after NACRT.

### Study objectives

#### Primary objective

The primary objective of the EXERT trial is to examine the effects of a supervised, high-intensity interval training (HIIT) program, compared to usual care, on cardiorespiratory fitness during NACRT.

#### Secondary objectives

The secondary objectives of the EXERT trial are to (1) compare an unsupervised, moderate-to-vigorous-intensity, continuous exercise training program after NACRT to usual care on cardiorespiratory fitness, (2) compare the supervised HIIT program during NACRT and unsupervised continuous exercise training program after NACRT on functional fitness, QoL, and symptom burden, (3) establish the feasibility and safety of the supervised HIIT program during NACRT, and (4) investigate the motivational outcomes and determinants of exercise during and after NACRT.

#### Exploratory objectives

The exploratory objectives of the EXERT trial are to compare a supervised HIIT program during NACRT followed by an unsupervised, moderate-to-vigorous-intensity, continuous exercise training program after NACRT to usual care on clinical outcomes including treatment toxicities, treatment completion, treatment response, and surgical complications.

## Methods/design

### Study design

**Fig. 2:**
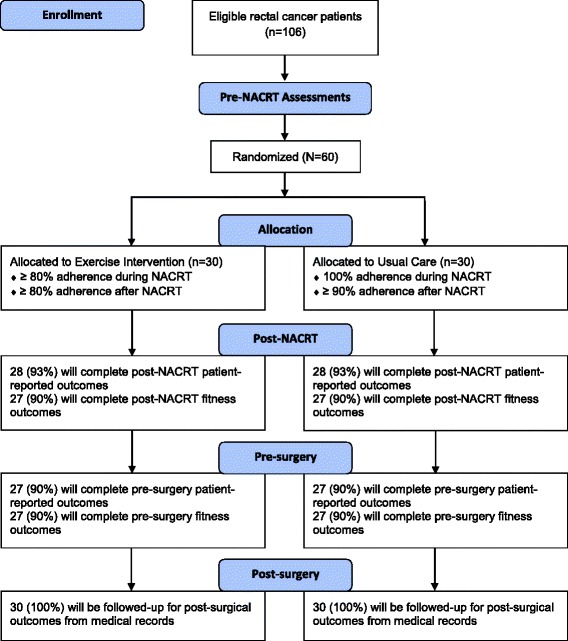
Proposed flow of participants through the Exercise During and After Neoadjuvant Rectal Cancer Treatment (EXERT) trial

**Fig. 3:**
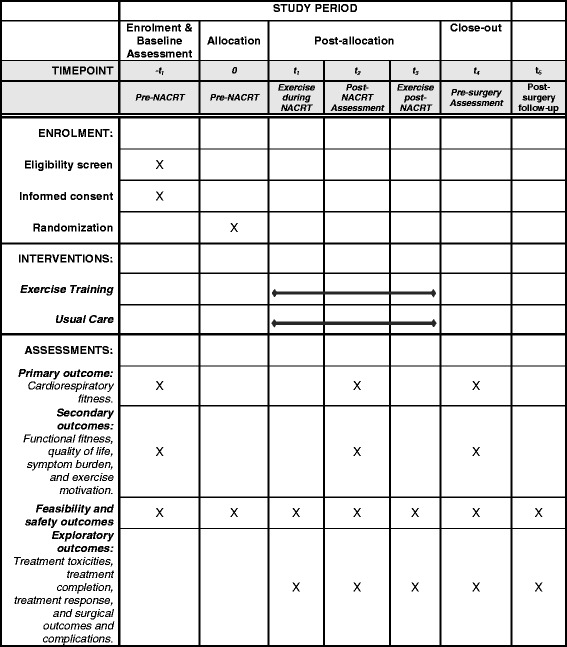
Standard Protocol Items: Recommendations for Interventional Trials (SPIRIT) Figure for the Exercise During and After Neoadjuvant Rectal Cancer Treatment (EXERT) trial

The EXERT trial will be a single-center, prospective, two-armed, phase II randomized controlled trial conducted in Edmonton, Alberta. The EXERT trial has been approved by the Health Research Ethics Board of Alberta-Cancer Committee and all participants will be required to provide written informed consent. The proposed participant flow through the study is shown in Fig. 2. Health-related fitness outcomes and patient-reported outcomes will be assessed at baseline (0–7 days before starting NACRT), post NACRT (0–7 days after completing NACRT), and pre surgery (7–14 days before the planned surgery date). The Standard Protocol Items: Recommendations for Interventional Trials (SPIRIT) Figure for the EXERT trial is shown in Fig. 3 (Additional file 1).

### Eligibility criteria

Men and women will be eligible for the trial if they (1) are ≥ 18 years old, (2) are scheduled to received standard NACRT consisting of 5–6 weeks of radiotherapy (45–54 Gy) with concurrent chemotherapy (orally administered capecitabine or intravenously administered 5-fluorarcil) followed by total mesorectal excision, (3) receive medical clearance to participate in the study as determined by their treating oncologist, the Physical Activity Readiness Questionnaire for Everyone (PAR-Q+), and a certified exercise physiologist, (4) are able to complete the pre-NACRT graded exercise test, (5) are not currently engaging in any regular vigorous-intensity exercise and/or ≥ 150 min of moderate-intensity-exercise/week, (6) are able to provided written informed consent and complete questionnaires in English, and (7) are willing to be randomized to exercise training or usual care (no exercise) for 12 weeks.

### Recruitment

Prospective patients will be approached by the treating radiation oncologist and study coordinator at the time of their initial radiation consultation. The study coordinator will follow-up with eligible patients by phone and schedule interested patients for pre-NACRT testing. This recruitment strategy was effective in our feasibility study with 18 of 32 patients (56%) being recruited over a 6-month period [16].

### Randomization and blinding

After completing all baseline assessments, patients will be randomly assigned to either the exercise training group or usual care group in a 1:1 ratio using block randomization. A research assistant, not otherwise involved in the trial, will generate the block sizes and randomization sequence using a computer-generated random allocation sequence which will be concealed from the recruiting study coordinator. Given the nature of the intervention, it is not possible to blind the investigators or participants to group allocation. Additionally, due to logistical challenges at our facility, it is difficult to blind outcome assessors to group allocation for the primary outcome of cardiorespiratory fitness and the secondary outcomes of functional fitness. Nevertheless, fitness outcome assessors will follow a detailed protocol and be trained in the importance of standardizing outcome assessments and avoiding bias. Moreover, outcome assessors will not review the metabolic data during the cardiorespiratory fitness test, and the criteria for achieving peak volume of oxygen consumption (VO_2_ peak) will undergo an independent review. Finally, outcome assessors will be blinded to group assignment for the exploratory outcomes of treatment toxicities, treatment completion, treatment response, and surgical complications which will be assessed by medical staff not otherwise involved in the study.

### Intervention

For patients randomized to the exercise training group, the intervention will be divided into two phases: (1) during NACRT and (2) post NACRT. During NACRT, all of the exercise sessions will be supervised by a certified exercise physiologist. We previously determined that it was feasible for patients to attend supervised exercise sessions at our fitness center (within a 5-min walk from the cancer center) since they were already coming to the cancer center 5 days/week for radiation treatment [16]. During NACRT, patients will be asked to complete 18 supervised HIIT sessions (i.e., three sessions/week for 6 weeks) and to continue with any light-to-moderate-intensity exercise that they were performing at baseline. We selected a HIIT program for evaluation because of its ability to maximize cardiorespiratory fitness improvements over a short period of time [18]. Moreover, HIIT has previously demonstrated safety and feasibility in clinical populations including patients with cardiometabolic disease [19], diabetes [20], and during adjuvant chemotherapy in patients with mixed cancers [21]. HIIT is characterized by relatively short bursts of vigorous-intensity exercise, interspersed by periods of rest or light-intensity exercise for recovery. There are an endless number of possible combinations that can make up a HIIT program; however, HIIT typically refers to exercise intensities corresponding to ≥ 85% of peak heart rate or ≥ 80% of VO_2_ peak [19]. We have designed the HIIT program in the EXERT trial to closely match a previously published HIIT program which has demonstrated feasibility, safety, and greater improvements in cardiorespiratory fitness in patients with coronary artery disease [22].

In our phase I study, we demonstrated an excellent median attendance rate of 83% to three sessions/week of moderate-intensity, continuous exercise training during NACRT [16]. Moreover, no adverse events were observed and our evaluation was that even higher-intensity exercise training would be feasible in this clinical setting. Nevertheless, the safety and feasibly of HIIT during NACRT in rectal cancer patients has yet to be established and is a key objective of our study.

In our feasibility study, the most frequently used modality was the treadmill (67.4% of sessions) [16]. Thus, the HIIT program will consist of uphill treadmill walking. Each HIIT session will start with a 5-min warm-up at a workload that elicits 30-40% of VO_2_ peak during the baseline graded exercise test. Patients will complete 2-min, high-intensity intervals at a workload that elicits 85% of VO_2_ peak during the baseline graded exercise test. Between the high-intensity intervals, the active-recovery intervals will consist of 2 min at a workload that elicits 40% of VO_2_ peak during the baseline graded exercise test. Each HIIT exercise session will end with a 5-min cool-down totaling 40 min/session. The number of HIIT intervals will begin at five and progress by one every second session up to eight intervals (Table 1).

**Table 1:** High-intensity interval training program during neoadjuvant chemoradiotherapy in the Exercise During and After Neoadjuvant Rectal Cancer Treatment (EXERT) trial

	Interval period			Recovery period			
Session	No.	Duration	Intensity	No.	Duration	Intensity	Total duration
		(min)	% VO_2_ peak^a^		(min)	% VO_2_ peak^a^	(min)
1–2	5	2	85	4	2	40	28
3–4	6	2	85	5	2	40	32
5–6	7	2	85	6	2	40	36
7–18	8	2	85	7	2	40	40

^a^Prescribed according to workload (treadmill speed and incline) that elicited 85% of VO_2_ peak (interval period) and 40% of VO_2_ peak (recovery period) during baseline graded exercise test

Note: All HIIT sessions start with a 5-min warm-up and end with a 5-min cool-down

Prior to each exercise session, an exercise specialist will assess blood pressure and heart rate and ask patients to report any immediate symptoms. Additionally, body temperature will be assessed in patients reporting any signs or symptoms of a fever. If body temperature is ≥ 38 °C, patients will be instructed not to exercise that day. For each supervised session, the exercise specialist will record attendance and the workload (i.e., treadmill speed and incline), Rating of Perceived Exertion (RPE) (Borg 0–10), and heart rate for each high-intensity interval. Optimal adherence to the supervised exercise sessions will be facilitated by scheduled appointments, flexibility in scheduling the exercise sessions (i.e., according to patients’ radiation sessions), immediate follow-up and re-booking of missed sessions, personable exercise trainers, and free parking. In our feasibility study, we identified the most common barriers to exercising during NACRT as side effects from chemoradiotherapy (88%), fatigue (76%), and diarrhea (71%) [23]. In the EXERT trial, we will optimize adherence to the supervised exercise sessions by modifying each session according to any immediate symptoms or side effects that patients are experiencing.

If a patient is experiencing immediate side effects that hinder their ability to complete the high-intensity intervals at the prescribed workload, the fitness attendant will modify the exercise session according to what the patient is able and willing to do. Options for modifying the exercise dose will include either reducing the workload of the high-intensity intervals, reducing the number of high-intensity intervals, or both. All reasons for dose modification will be noted.

After NACRT, patients will be asked to complete at least 150 min of unsupervised, moderate-to-vigorous-intensity, continuous exercise training training/week (current Canadian Physical Activity Guidelines). In our phase I study, it was feasible for patients to achieve 150 min of mostly unsupervised, moderate-intensity exercise/week after NACRT. Although local patients in our pilot study were offered a supervised exercise program after NACRT, only two out of 16 patients expressed interest in continuing with supervised exercise in this phase and patients mainly achieved their weekly exercise minutes by walking outdoors or by using pre-existing home exercise equipment (e.g., treadmill, elliptical, and upright bike). For this reason, we felt that it would be difficult to deliver a standardized and replicable HIIT program after NACRT. Moreover, in our phase I study, cardiorespiratory fitness improved by 2.4 ml/kg/min from post NACRT to pre surgery suggesting that an unsupervised, moderate-intensity, continuous exercise training program may be effective at improving cardiorespiratory fitness after NACRT and prior to surgery in rectal cancer patients. Finally, after our experience in the phase I study, we felt that it would be feasible and safe for rectal cancer patients to complete moderate-to-vigorous-intensity continuous exercise in this phase and may result in greater improvements in cardiorespiratory fitness. Nevertheless, one of the goals of the EXERT trial is to further establish the feasibility and safety of continuous exercise training after NACRT and to determine its preliminary efficacy at improving outcomes for rectal cancer patients.

After NACRT, the exercise will be individualized according to patients’ post-NACRT graded exercise test (i.e., heart rate that corresponded with approximately 46–91% of VO_2_ peak) [24]. Patients will be provided with a heart rate monitor and will also be instructed on how to use RPE and the talk-test to determine the intensity of their exercise sessions. Patients will be provided with examples of how to complete the exercise (e.g., 30 min, 5 days/week; 50 min, 3 days/week). Moreover, patients will be instructed that the exercise completed in this phase should be in addition to what they were already doing at baseline. Finally, patients will receive printout materials with instructions on how to complete the exercise in the post-NACRT phase as well an exercise log to help them keep track of their exercise. After NACRT, optimal adherence will be achieved using a more formal behavioral support program based on the Theory of Planned Behavior (TPB) [25]. The study coordinator will maintain weekly contact with each patient via telephone and offer behavioral support sessions consisting of standard behavioral change techniques including goal setting, planning, self-monitoring, and overcoming barriers.

#### Usual care group

Patients randomized to the usual care group will receive standard medical care which includes meeting with a dietician weekly to ensure adequate caloric and nutrient intake. Patients in the usual care group will be asked not to increase their physical activity/exercise levels during or after NACRT. Exercise is not currently part of standard care for these patients at our center and patients do not receive any exercise recommendations. After the pre-surgery assessment, patients in the usual care group will be offered a copy of the Canadian Physical Activity Guidelines and encouraged to initiate an exercise program after they recover from surgery and receive medical clearance from their physician.

### Outcome measures

#### Primary outcome measure

We selected *cardiorespiratory fitness* as the primary endpoint for the EXERT trial because there is clinical equipoise as to whether 6 weeks of HIIT during NACRT is sufficient to meaningfully improve cardiorespiratory fitness. Moreover, cardiorespiratory fitness is an established surrogate for some patient-reported outcomes and clinical outcomes [9, 26–29]. Our primary measure of cardiorespiratory fitness, VO_2_ peak, will be assessed by the modified Bruce graded exercise test on a treadmill with direct measures of cardiorespiratory variables using a metabolic measurement system (Parvo Medics TrueOne® 2400; Sandy, UT, USA ) [30, 31]. The modified Bruce treadmill protocol was designed for use in high-risk and elderly individuals. Briefly, the protocol will start at 1.7 mph and 0% grade and will progress every 3 min until the patient reaches volitional fatigue or if any exercise contraindications occur. During the test, heart rate will be monitored continuously and recorded every minute and blood pressure, oxygen saturation, and rating of perceived exertion will be measured and recorded in the last minute of every stage. Immediately after the test, patients will complete a 5-min active recovery (1.7 mph and 0% grade). During the active recovery, heart rate, blood pressure, and oxygen saturation will continue to be monitored and recorded at 1 min and 5 min.

#### Secondary outcome measures

*Functional fitness* will be assessed by the Senior’s Fitness Test which measures basic mobility-related parameters associated with functional abilities in the everyday living of older adults [32, 33]. The Senior’s Fitness Test consists of six items including the 30-s chair stand (assessment of lower body strength), the arm curl (assessment of upper body strength), the chair sit-and-reach (assessment of lower body flexibility), the back scratch (assessment of upper body flexibility), the 8-foot up-and-go (assessment of agility and dynamic balance), and the 6-min walk (assessment of aerobic endurance).

*Quality of life* will be assessed by the widely used and validated European Organisation of Research and Treatment of Cancer (EORTC) core 30-item questionnaire (QLQ-C30) version 3.0 [34]. We selected the EORTC QLQ-C30 because it assesses symptoms, physical function, psychosocial function, and overall QoL [35]. The EORTC QLQ-C30 is composed of five multi-item functional scales (physical, cognitive, role, emotional, and social), three multi-item symptom scales (fatigue, nausea and vomiting, and pain), five single-item symptom scales (dyspnea, insomnia, loss of appetite, constipation, and diarrhea), a single-item financial impact scale, and a two-item global health and QoL scale. Additionally, the EORTC-QLQ-CR29 (colorectal cancer) will be used to assess QoL. The EORTC QLQ-CR29 has demonstrated acceptable validity and reliability for its supplemental use with the QLQ-C30 to assess the QoL of colorectal cancer patients during treatment [36]. The EORTC QLQ-CR29 contains 29 questions and evaluates urinary dysfunction, gastrointestinal symptoms, body image, separate concerns for persons with or without a stoma, and sexual function (separate scale for men and women). Items for both questionnaires are evaluated using a 1-week time frame (i.e., “during the past week”) on a 4-point scale (“not at all,” “a little,” “quite a bit,” or “very much”), except for the global health scale of the QLQ-30, which is measured on a 7-point scale ranging from “very poor” to “excellent.” For both the QLQ-C30 and QLQ-CR29, a higher score on the functional scales indicates better functioning, whereas a higher score on the symptom scales indicates worse symptoms.

*Overall symptom burden* will be assessed by the M.D. Anderson Symptom Inventory (MDASI) [37]. We selected the MDASI because it is brief and easy to use, and captures the most frequently reported disease- and treatment-related symptoms. The MDASI scale consists of 13 core symptom items (pain, fatigue, nausea, disturbed sleep, distress (emotional), shortness of breath, lack of appetite, drowsiness, dry mouth, sadness, vomiting, difficulty remembering, and numbness or tingling) and six interference items (general activity, mood, walking ability, normal work, relations with other people, and enjoyment of life). In addition to the 13 core symptoms, we will incorporate four additional symptoms that are specifically relevant in this clinical setting: mouth sores, hand-foot syndrome, diarrhea, and skin reaction at the site of irradiation.

*Exercise motivation* will be assessed using the TPB [25]. The key TPB constructs including attitudes, subjective norms, intention, and perceived behavioral control will be assessed using standardized items [38]. At the pre-NACRT time point, all patients will be asked to prospectively evaluate their motivation for the HIIT program. After NACRT, patients randomized to the exercise group will be asked to retrospectively evaluate their motivation for the HIIT program during NACRT and their prospective motivation for the exercise program post NACRT. Before surgery, patients in the exercise group will be asked to evaluate their retrospective motivation for the exercise program post NACRT, and all patients will be asked to evaluate their prospective motivation for exercising after surgery. All questions will be evaluated on a 5-point scale ranging from 1 (“not at all”) to 5 (“very much”).

#### Feasibility and safety

The feasibility and safety of the exercise intervention will be determined based on eligibility rate, recruitment rate, exercise adherence rate, assessment rate, and adverse events. The willingness of rectal cancer patients to be randomized to a supervised HIIT program during NACRT is unknown; however, based on the results of our feasibility study, we anticipate a recruitment rate ≥ 50% [16]. Moreover, we do not know the willingness of patients in the usual care group to return for all follow-up assessments; however, based on the results of our feasibility study, we anticipate a follow-up assessment rate ≥ 80% at each time point [16].

Exercise adherence during NACRT will be assessed by the number of exercise sessions attended out of 18 as well as adherence to the workload and duration of the high-intensity intervals. Based on the results from our phase I study [16], we anticipate a median attendance rate to the supervised exercise training during NACRT ≥ 80%. Exercise adherence to the unsupervised exercise training after NACRT will be assessed by self-report using the Godin Leisure-Time Exercise Questionnaire (GLTEQ) [39]. Based on our previous results [16] we anticipate that the mean number of moderate-to-vigorous-intensity exercise minutes post NACRT will be ≥ 222 per week. Safety will be assessed by monitoring any serious adverse events that occur during exercise testing or the supervised exercise sessions. No serious exercise-related adverse events were observed in our previous study [16].

#### Exploratory outcome measures

*Treatment toxicities* will be assessed by clinical nurses on a weekly basis during NACRT using the National Cancer Institute Common Toxicity Criteria for Adverse Events Version 3.0. *Treatment completion* will be assessed as the number of patients completing 100% of their planned radiation dose within 1 week of the planned completion date using electronic medical records. Additionally, the number of patients receiving ≥ 80% of their planned chemotherapy dose will be recorded. *Treatment response* will be assessed by pathologic complete response as reported by the pathologist after reviewing the surgical sample. *Surgical outcomes and complications* will be obtained from medical records.

#### Baseline descriptive variables

*Demographic variables* will be collected from the baseline questionnaire and will include age, sex, marital status, education, income, employment status, and ethnicity*. Behavioral variables* will include smoking and physical activity. Physical activity (PA) will be assessed using the GLTEQ [39]. All participants will complete the GLTEQ at baseline (PA in the past month), post NACRT (unsupervised PA in the exercise training group; all PA in the usual care group), and pre surgery (all PA in both the exercise training and usual care groups). *Medical variables* will be abstracted from medical records at baseline and will include disease stage, chemotherapy protocol, and ostomy. Comorbidities and a list of medications will be collected in the baseline questionnaire.

*Body composition/anthropometry* will be assessed by height, weight, and waist and hip circumference [40, 41].

### Sample size

Based on our feasibility study recruiting 18 patients in 6 months, we anticipate recruiting 60 patients over a 20-month period and randomizing 30 patients to each group. Based on this sample size, our study has 80% power, with a two-tailed alpha < 0.05, to detect a clinically meaningful effect of 3.5 ml/kg/min on our primary outcome of VO_2_ peak post NACRT, assuming a standard deviation of 5.6 ml/kg/min, 10% missing data, and adjustment for baseline value and other prognostic covariates [42]. This power may be sufficient for detecting differences in our secondary patient-reported outcomes if the effects are at least moderate (i.e., a standardized effect sizes of approximately ≥ *d* = 0.60). This power is unlikely sufficient for detecting potentially meaningful differences in any of the exploratory clinical outcomes. Given that the purpose of this phase II trial is to inform phase III trials, the patient-reported and clinical outcomes will be interpreted for potential clinical significance based on the direction and magnitude of numerical differences.

### Data analysis

We will use analysis of covariance (ANCOVA) at both the post-NACRT and pre-surgery time points to compare the two groups on all primary and secondary outcomes with adjustment for baseline value of the outcome as well as other potential covariates. All statistical analyses will be based on the intention-to-treat principle and include all patients with baseline and follow-up data. No missing data replacement strategies will be performed for this phase II trial as we anticipate < 10% missing data. Chi-square analyses will be used to explore between-group differences in the categorical and ordinal clinical outcomes. All analyses will be performed using SPSS (SPSS Inc., Chicago, IL, USA).

## Discussion

NACRT is part of standard care for locally advanced rectal cancer and results in improvements in local recurrence rates and surgical outcomes. Furthermore, about 10–20% [43, 44] of patients achieve a pathologic complete response to NACRT which is associated with better disease control and surgical outcomes [45]. Moreover, despite advances in supportive care management, NACRT still causes toxicities that can negatively impact outcomes for rectal cancer patients. Interventions to manage side effects, improve QoL and optimize treatment outcomes are needed. Exercise is a low-cost, low-toxicity intervention that improves symptom management, cardiorespiratory fitness, and QoL in several cancer patient groups; however, no definitive studies have examined the impact of exercise on outcomes for locally advanced rectal cancer patients. Loughney et al. [15] are currently conducting a randomized controlled trial in rectal cancer patients focused on the post-NACRT phase and Moug et al. (ISRCTN62859294) are currently examining the effects of an unsupervised walking program during and after NACRT. We propose that an exercise training intervention both during and after NACRT may have additional benefits for symptom management, QoL, treatment outcomes, and possibly even survival (Fig 1).

The results from our phase I, single-arm study demonstrated that rectal cancer patients are willing and able to participate in a supervised, moderate-intensity, continuous exercise training intervention during NACRT followed by an unsupervised, moderate-intensity, continuous exercise training intervention after NACRT [16]. More specifically, we reported an excellent recruitment rate of 56% (18/32 patients) over 6 months and a follow-up assessment rate of > 80% [16]. Moreover, the median attendance rate for the supervised exercise during NACRT was 83%. After NACRT, patients completed an average of 222 ± 155 min/week of unsupervised exercise [16]. No adverse events were observed and our evaluation was that even higher-intensity exercise would be feasible in this clinical setting [16]. Despite the exercise intervention, most health-related fitness outcomes and patient-reported outcomes declined during NACRT and recovered after NACRT [16]. Consequently, any benefit from exercise during NACRT is likely related to preventing declines in functioning. Moreover, patients reported that they experienced several benefits from exercise (e.g., physical fitness, QoL, self-esteem) but they also perceived some potential harms (e.g., worsening fatigue, diarrhea, skin irritation, hand-foot syndrome) [23]. We concluded that moderate-intensity, continuous exercise training during and after NACRT for rectal cancer is feasible and safe, and that phase II randomized controlled trials are needed to establish the benefits (and possible harms) of exercise in this clinical setting [16, 23].

The EXERT trial will be the first to evaluate the efficacy of exercise training for improving outcomes in rectal cancer patients during and after NACRT. Furthermore, the EXERT trial will establish the feasibility and safety of a HIIT program in this unique and challenging clinical setting. To date, most exercise oncology studies have focused on high-volume, continuous, moderate-to-vigorous-intensity exercise training [46]. HIIT is receiving attention in cancer patients [47–49] because of its ability to generate larger and more rapid improvements in maximal volume of oxygen consumption (VO_2_ max) which may be a surrogate for important clinical outcomes such as QoL and survival [9, 26, 28]. Although moderate-intensity, continuous exercise training is beneficial for cancer patients, HIIT may be viewed as a potentially “high-risk, high-reward” exercise training intervention because its greater risk for safety and feasibility challenges may be offset by its greater potential for improved outcomes. Moreover, HIIT may be especially attractive in clinical settings, such as during NACRT or pre surgery, where shorter time frames are available for intervention delivery.

The EXERT trial will also be one of the few exercise oncology trials to examine the impact of exercise in the neoadjuvant setting and one of the few to include clinical cancer outcomes (e.g., treatment completion, pathologic complete response, post-surgical outcomes). In the rectal cancer setting, observational data suggest that cardiorespiratory fitness declines during NACRT and that pre-surgical cardiorespiratory fitness may predict post-surgical complications [29]. Initiating an exercise training intervention during NACRT could potentially optimize improvements in cardiorespiratory fitness which may result in fewer post-surgical complications and better post-surgical recovery when compared to an exercise training intervention initiated after NACRT. Additionally, poor compliance to external-beam radiation has been associated with an increased risk of disease recurrence and death [50]. If exercise is effective in managing symptoms and subsequently improving treatment compliance, radiation therapy may be optimized and result in better outcomes in this clinical setting. Finally, tumor hypoxia has been identified as a factor limiting the effectiveness of radiation therapy [51]. Pre-clinical models suggest that exercise may cause favorable changes in the vasculature of solid tumors thereby enhancing tumor oxygenation and possibly the effectiveness of radiation therapy [52–55]. Although these findings are intriguing, they have yet to be replicated in human clinical trials. Nevertheless, these are the outcomes that are most important to patients and clinicians and likely to drive changes in clinical practice.

To summarize, EXERT is the first phase II trial designed to generate preliminary efficacy data on the benefits and harms of exercise training, including clinical outcomes, in rectal cancer patients during and after NACRT. Additionally, EXERT will also establish the feasibility and safety of a supervised HIIT program in rectal cancer patients during NACRT. If the EXERT trial shows that exercise is safe, tolerable, and produces meaningful improvements in cardiorespiratory fitness, symptom management, QoL, and/or clinical outcomes, larger phase II and III trials designed to target these outcomes will be necessary to determine if exercise should be integrated in standard clinical care for this patient population.

### Trial status

The trial opened for accrual in June of 2017 and is expected to be completed by June of 2019.

## Additional file

Additional file 1: SPIRIT 2013 Checklist: recommended items to address in a clinical trial protocol and related documents. (DOC 120 kb)

## Abbreviations

EORTC: European Organisation of Research and Treatment of Cancer
GLTEQ: Godin Leisure-Time Exercise Questionnaire
HIIT: High-intensity interval training
MDASI: M.D. Anderson Symptom Inventory
NACRT: Neoadjuvant chemoradiotherapy
PA: Physical activity
QoL: Quality of life
TPB: Theory of Planned Behavior
VO_2_ peak: Peak volume of oxygen consumption
